# Hysterectomy by transvaginal natural orifice transluminal endoscopic surgery: An Italian initial experience

**DOI:** 10.3389/fmed.2022.1018232

**Published:** 2022-12-13

**Authors:** Maria Lieta Interdonato, Paolo Scollo, Tommaso Bignardi, Francesca Massimello, Martina Ferrara, Gianluca Donatiello, Marta Caretto, Paolo Mannella, Basilio Pecorino, Mario Giuseppe Meroni, Tommaso Simoncini

**Affiliations:** ^1^Department of Obstetrics and Gynecology, Niguarda Hospital, Milan, Italy; ^2^Department of Obstetrics and Gynecology, Cannizzaro Hospital, Catania, Italy; ^3^Department of Clinical and Experimental Medicine, University of Pisa, Pisa, Italy

**Keywords:** natural orifice transluminal endoscopic surgery (NOTES), vNOTES, hysterectomy, laparoscopy, vaginal surgery, endoscopic surgery

## Abstract

**Background:**

The aim of this study is to evaluate the initial feasibility, safety, and outcomes of hysterectomy performed by transvaginal natural orifice transluminal endoscopic surgery (vNOTES) at three institutions in Italy.

**Materials and methods:**

All women who underwent vNOTES hysterectomy ± salpingo-oophorectomy for benign indications at three tertiary referral medical centers between July 2019 and April 2021 were included in a retrospective analysis. All vNOTESs were performed with the use of Alexis^®^ and Vpath Gel paths^®^ (Applied Medical). Perioperative data were extracted from patient records. Patient satisfaction and dyspareunia were prospectively inquired about at 60 days and 6 months.

**Results:**

Forty-six patients underwent vNOTES in the study period. Indications for surgery included myomas ± metrorrhagia (52.2%), H-Sil/*in situ* cervical cancer (10.7%), adenomyosis ± metrorrhagia (8.7%), BRCA 1-2 mutations (6.5%), endometrial hyperplasia (6.5%), ovarian cyst + history of breast cancer (6.5%), metrorrhagia (6.5%), and hydatidiform mole (2.2%). The mean operation time was 91.1 (±32.6) minutes. The mean hemoglobin drop was 1.2 (±0.8). The mean visual analog scale at 24 h for post-operative pain was 3.3 (±1.8). Secondary to our limited experience with the surgical technique, we favor discharge only from day 1. The mean length of hospital stay was 2 (±1.4) days. Two conversions to conventional laparoscopy were reported (4.3%), due to an obliterated pouch of Douglas and a preoperative complication. Two post-operative complications were reported (4.3%). Overall, our data on peri- and post-operative outcomes are similar to those already published for vNOTES.

**Conclusion:**

Our initial experience suggests that introducing vNOTES as an alternative to conventional surgery is feasible and may offer some advantages in selected women.

## Introduction

Laparoscopic procedures have been used in minimally invasive gynecological surgery since the late 1980s. Nowadays, laparoscopic surgery has become the gold-standard approach for most benign and malignant gynecological diseases (1). Laparoscopy has many advantages compared to laparotomy, including a shorter operation time, better cosmetic effects, fewer adhesion formations, fewer abdominal wall infections, fewer febrile episodes, less post-operative pain, and a shorter recovery (2, 3).

A very attractive feature in gynecological surgery is the possibility to use the vagina to perform natural orifice transluminal endoscopic surgery (NOTES) to avoid entering through the abdomen to gain access to pelvic structures. Surgical procedures through natural orifices (NOTES) such as the mouth, vagina, urethra, and rectum were first described by Kalloo et al. (4) in 2004 in a porcine model. Then, Reddy and Rao (5) performed the first transgastric appendectomy in humans using a flexible endoscope, which ignited worldwide interest in the NOTES technique.

Compared to other natural orifices, the transvaginal route for NOTE appears to be preferable, permitting a safe entry and simple closure. The vaginal route has quickly surpassed other transluminal access routes and is currently adopted in various surgical procedures, such as cholecystectomy, appendectomy, sigmoidectomy, nephrectomy, splenectomy, and more recently liver resection and sleeve gastrectomy in bariatric surgery (6).

Transvaginal NOTES (vNOTES) applied to gynecological conditions is a combination of conventional vaginal surgery and laparoscopic single-port surgery. In the last decade, the use of the vNOTES approach to perform gynecological surgery has been increasingly reported (7–11). vNOTES is a safe, effective, and scarless technique that can overcome the limitations of poor visualization and manipulation related to vaginal surgery. When compared to standard laparoscopy, the use of vNOTES may be associated with less post-operative pain, faster post-operative recovery, shorter operative times, and an improvement in cosmetic results (7, 12).

In this article, we would like to report our experience with the first introduction of vNOTES in three gynecological centers in Italy, with a focus on feasibility, perioperative outcomes, safety, and patient satisfaction with this technique.

## Materials and methods

We conducted a multicentric retrospective analysis of the first 46 cases of vNOTES procedures performed at three different institutions: Grande Ospedale Metroplitano Niguarda of Milan, Ospedale Santa Chiara of Pisa, and Ospedale Cannizzaro of Catania. vNOTES were performed for benign gynecological indications between July 2019 and May 2021. All surgeries were performed by five surgeons at the three centers (MM, MI, TS, PM, and PS). vNOTES surgery was not offered to women with suspected pelvic adhesions and/or endometriosis upon pelvic examination, the previous history of pelvic inflammatory disease, virginity, and contraindication to general anesthesia, pneumoperitoneum, or Trendelenburg position. Perioperative data were collected retrospectively from hospital electronic databases and patient files. The following data were collected: age, body mass index (BMI, kg/m^2^), number of vaginal deliveries and C-sections, previous pelvic and abdominal surgery, indication for operation, type of surgery, total operation time (defined as the time between the incision of the vagina and the closure of the vaginal cuff), intraoperative and post-operative complications, estimated blood loss, serum hemoglobin (Hb) drop (24-h difference between preoperative and post-operative hemoglobin), post-operative pain using the visual analog scale (VAS) score at 6 and 24 h, and length of post-operative hospital stay. All post-operative complications were recorded: any surgical procedure failure, pelvic visceral injury, blood transfusion, vaginal vault bleeding or infection, urinary tract infection, or post-operative fever. Patients were advised to avoid vaginal intercourse for at least 6 weeks after surgery and were scheduled for a post-operative follow-up at 60 days and 6 months. Patient satisfaction and dyspareunia were inquired prospectively at 60 days and 6 months.

### Surgical technique

Each surgeon started to perform vaginally assisted NOTES hysterectomy with or without salpingectomy/salpingo-oophorectomy. Hysterectomy procedures through vNOTES have been standardized by Baekelandt et al. (12).

Surgery was performed with the patient in a lithotomy position associated with a slight Trendelemburg. An 18F bladder catheter was inserted to empty the bladder. The perineum and vagina were disinfected using a 10% povidone-iodine solution. General anesthesia or combined general plus spinal anesthesia was used. Preemptive analgesia with paracervical blocks was performed in all women: a saline solution with 0.9% adrenaline and ropivacaine was injected into the vaginal mucosa and uterosacral ligaments. vNOTES hysterectomy involves three surgical steps:

#### First vaginal step

A standard circular incision around the uterine cervix and dissection of the vaginal mucosa were performed. The posterior vaginal fornix was visualized and dissected, and the pouch of Douglas was opened. The uterosacral ligaments were identified, clamped, incised, and sutured. Then, the vescicouterine space was dissected to expose the vescicouterine pouch and the peritoneum was opened. A vaginal port (Gelpoint^®^, Applied Medical, Rancho Santa Margarita, CA, USA) with Alexis^®^, (Applied Medical) was installed, and CO_2_ was insufflated to create a low-pressure pneumoperitoneum (8–10 mm Hg).

#### Second endoscopic step

Three 10-mm trocars were used in all cases. A standard rigid 0- or 30-degree camera, bipolar forceps, and a sealing device were used. After achieving a sufficient pneumoperitoneum, the broad ligaments and transverse cervical fascia were exposed bilaterally and dissected with the vessel sealer device. The ligaments were sealed and cut near the cervix and uterus to prevent ureteral injury. The uterine and ovarian vessels were sealed and cut stepwise with the sealing device when salpingo-oophorectomy was performed. In the case of bilateral salpingectomy, the utero-ovarian ligaments and the mesosalpinges were sealed. After ensuring hemostasis, the pneumoperitoneum was deflated and the port device was removed to extract the uterus through the vagina, under the protection of the vaginal retractor. We performed cold knife morcellation when the size of the uterus hinders transvaginal specimen extraction.

#### Third vaginal step

The vaginal cuff was closed using two coated vicryl sutures (90 cm, polyglactin 910; Ethicon Endo-Surgery, Norderstedt, Germany).

Pre- and intraoperative care were guided as per the ERAS protocol for gynecological procedures, which was implemented at the three centers before this study. With regards to post-operative analgesia, we used paracetamol 1 g i.v. q.i.d. on day 1, with Ketorolac 30 mg i.v. as a rescue. In the three centers involved, different antibiotic prophylaxis schemes had been used, to follow the different hospitals’ protocols. We gave all patients in Milan an antibiotic prophylaxis cefazolin 2 g i.v. and Metronidazole 500 mg 30 min before surgery, plus cefazolin 2 g i.v. and Metronidazole 500 mg as a single dose 8 h after surgery. Cefazolin 2 g iv 30 min before surgery was given in Pisa, while amoxicilline + clavulanic acid 2 g i.v. 30 min before surgery were given in Catania.

## Statistical analysis

GraphPad Prism 7 (GraphPad Software Inc., San Diego, California, USA) program was used for data analysis. The parameters assessed in women have been included in the tables. Category variables have been represented as frequencies and percentages, while continuous variables were represented as averages and standard deviations (SDs).

We compared data observed in our experience to other previous published retrospective data: ordinary or repeated measures (RM) one-way ANOVA, followed by paired sample Tukey’s multiple comparisons test was performed when comparing operative time, Hb drop, VAS, and length of stay, meanwhile Chi-square test was performed when comparing post-operative complication rate and conversion rate.

For all comparisons, the values of *p* < 0.05 were considered statistically significant.

## Results

Forty-six patients underwent vNOTES surgery in the study period. In all cases, a vNOTES hysterectomy was performed. The patient’s characteristics and indications for surgery are summarized in Table 1. Thirty-three (71.7%) women had at least one vaginal delivery, six patients (13%) had a cesarean section and two patients (4.3%) had two previous cesarean sections. Table 2 shows the patient’s surgical outcome. The mean operation time was 91.1 min. The mean drop in the Hb level was 1.2 g/dl. No patient needed a post-operative blood transfusion. Conversion to conventional laparoscopy was needed in two patients (4.3%). In one case, the conversion was secondary to the inability to enter the posterior peritoneum vaginally: the subsequent laparoscopy showed obliteration of the pouch of Douglas due to severe endometriosis, which was not diagnosed preoperatively. In the second case, the surgeon decides to perform a total laparoscopic hysterectomy (TLH) instead vNOTES hysterectomy, because of pre-operatory ultrasounds evidence of hemoperitoneum associated with a Hb drop of 3 g/dl. No laparotomic conversion was needed.

**TABLE 1:** Population features analysis and description (*n* = 46).

Population (*n* = 46)	
Age (mean ± SD)	51 ± 7.2
BMI kg/m^2^ (mean ± SD)	24.4 ± 3.5
Hb level (g/dl) (mean ± SD)	12.6 ± 1.6
Parity (mean ± SD)	1.3 ± 1.0
C-section (mean ± SD)	0.1 ± 0.4
Previous abdominal surgery (mean ± SD)	0.8 ± 0.7
**Indications for surgery (*n*, %)**	
Myomas ± metrorrhagia	24 (52.2%)
H-Sil/*In situ* cervical cancer	5 (10.7%)
Adenomyosis ± metrorrhagia	4 (8.7%)
BRCA 1/2 mutation	3 (6.5%)
Endometrial hyperplasia	3 (6.5%)
Ovarian cyst + history of breast cancer	3 (6.5%)
Metrorrhagia	3 (6.5%)
Hydatiform mole	1 (2.2%)

SD, standard deviation.

**TABLE 2:** Perioperative and post-operative outcome of vNOTES in our case series, and comparison with previous studies by Beakelant et al. (14), Su et al. (16), Lee et al. (15), and Wang et al. (13).

	Italian group (*n* = 46)	Baecklendant et al. (14) (*n* = 10)	Su et al. (16) (*n* = 16)	Lee et al. (15) (*n* = 137)	Wang et al. (13) (*n* = 20)
Operative time (mean ± SD)	91.1 (32.6)	97 (23.8) *p* = ns	122.7 (17.6) *p* < 0.001	88.2 (4.1) *p* = ns	86.3 (23.7) *p* = ns
Uterine weight (mean ± SD)	250.39 (209.1)	51 (358) *p* = 0.02	538.8 (102.9) *p* = 0.0001	450.0 (24.1) *p* = 0.0001	352 (159) *p* = ns
Hb drop 24 h (mean ± SD)	1.2 (0.8)	1.5 (0.6) *p* = ns	1.9 (1.8) *p* = 0.001	1.2 (0.1) *p* = ns	–
VAS score 24 h (mean ± SD)	3.3 (1.8)	1.7 (0.5) *p* = 0.007	–	–	–
Leigh of stay (mean ± SD)	2.0 (1.4)	3 (0) *p* < 0.001	2.8 (0.2) *P* < 0.001	2.8 (0.1) *p* < 0.001	2.2 (0.5) *p* = ns
Post-operative complication (*n*, %)	2 (4.3%)	2 (20%) *p* = ns	0 *p* = ns	5 (3.6%) *p* = ns	0 –
Conversion rate (*n*, %)	2 (4.3%)	0 *p* = ns	0 *p* = ns	7 (5.1%) *p* = ns	1 (5%) *p* = ns

The data were analyzed statistically by RM-one-way-ANOVA, followed by Tukey’s multiple comparisons and Chi-square test (*p* < 0.05).

All women had very low post-operative pain scores, with a mean post-operative VAS pain score of 3.3 at 24 h. In six (13%) patients, the VAS score was not recorded.

The mean post-operative length of stay was 2 days. Of the 46 patients, 13 (28.2%) were discharged the first day after surgery; meanwhile, 17 (36.9%) were discharged 2 days after surgery. Of these patients, 12 (26.1%) declared to be able to go home the same day. Subsequently, five patients (10.9%) were treated in day surgery. Ten patients (21.7%) were hospitalized for 3–5 days.

Overall, we reported two post-operative complications (4.3%). In one case, the patient developed left ureteral kinking and was readmitted 3 days after surgery for left renal colic. At the same day, she went to a second surgery for removing vaginal vault sutures, left ureteral stent positioning, and colporrhaphy. In the second case, the complication was not related to the surgical procedure. The patient developed post-operative pneumonia, and she was discharged in good clinical condition after 7 days of parental antibiotic therapy.

The pathological examinations confirmed benign conditions in all specimens. At the 60-day follow-up visits, we did not notice any sign of vaginal infection or other problems related to vaginal scar healing. At 6-month follow-up in all patients, we observe the complete healing of the vaginal scar, and no patient reported pelvic pain or dyspareunia.

## Discussion

To the best of our knowledge, this is the first report about the introduction of vNOTES from gynecological centers based in Italy. In the last few years, surgeons have been trying to find the right place for vNOTES in gynecological surgery, with several experiences with this technique being increasingly reported from all over the world (7–11). Although the vNOTES non-inferiority compared to laparoscopy has been proved (7), there is still an international debate about vNOTES indications and potential advantages compared to other traditional techniques. Notwithstanding this, since the device’s approval in 2018, an increasing number of gynecological surgeons have already started introducing vNOTES in their practice. Altogether, we could say that introducing vNOTES in our three centers has been successful, in terms of feasibility, surgical outcomes, and safety. Wang et al. (13) retrospectively reviewed the first 240 vNOTES hysterectomy procedures performed by a single surgeon, identifying four phases in the vNOTES learning curved. In order to evaluate the efficiency of the introduction of vNOTES in our centers, we compared our initial experience with the outcome observed in Wang’s first phase of the learning curve (initial learning phase) and to other previously published data on vNOTES initial experiences in other centers (13–16). Our results in terms of operative time, Hb drop, length of stay, conversion rate, and post-operative complication rate were superimposable compared to other previously published data about vNOTES initial experience (Table 2) (13–16). Thus, vNOTES proved to be a safe approach, with short operating times, low post-operative pain, and short length of stay. We considered that sharing also our initial experience could help understand common issues with vNOTES initial adoption and reproducibility.

A potential issue about vNOTES regard the prolongation of operative time. Although, a previous review reported a statistically significant reduction of operative time for vNOTES hysterectomy compared to TLH, laparoscopic-assisted vaginal hysterectomy (LAVH), and single-port laparoscopic-assisted vaginal hysterectomy (12).

From our initial experience, we observe short mean operative (Table 2), in line with the one reported by Beackeladt et al. (14), Lee et al. (15), and Wang et al.(13), and significantly lower compared to the one reported by Su et al. (16). It worth to notice how the mean specimen weight in our case series is significantly lower than the one reported in the studies by Lee et al. (15) and Wang et al. (13) but significantly higher than that reported by Beackeladt et al. (14).

Another important feature of vNOTES is that it provides safe entry, easy access, and direct vision of the peritoneal cavity (17). Differently from TLH, LAVH, or single-site laparoscopic surgery, when using vNOTES, there is no risk related to blind trocar insertion, no risk of post-operative hernia, infection, hematoma, or adhesion formation at the trocar sites. Moreover, VNOTES is associated with better cosmetic results (9, 15, 17). Despite complications related to the first trocar entry occurring in less than 1% of the patients (18), when they do happen, vascular or visceral organ injuries, morbidity, and mortality increase markedly. In our small case series, vNOTES has helped avoid the risk of trocar entry complications in two women who had previously undergone extensive abdominal surgery, including liver–kidney transplant and previous laparocele correction. During vNOTES, we confirmed the presence of extensive adhesions in the upper abdomen; thus, we realized that the choice of the VNOTES approach was crucial.

However, the first vaginal step of vNOTES surgery, as in conventional vaginal hysterectomy, has been described as the most challenging gone, secondary to the risk of bladder or rectal lesion (14, 15). Therefore, a good amount of experience in vaginal surgery should be regarded as a fundamental prerequisite if you want to perform vNOTES surgery for the first time. In our centers, vNOTES hysterectomies were performed by surgeons skilled in vaginal surgery and we did not observe any bladder or rectal injury during the first vaginal step.

From a technical point of view, in our initial experience, one case of conversion to transabdominal laparoscopy was secondary to the failed attempt to perform colpotomy for a severe pouch of Douglas obliteration caused by unknown endometriosis. Also, Lee et al. (15) reported a case of conversion due to cul-de-sac obliteration; meanwhile, Wang et al. (13) reported one case of conversion secondary to infundibulopelvic ligament bleeding. No case of laparoscopic conversion was reported by Baekelandt et al. (14) and Su et al. (16), likewise in another subsequent trial (7, 9).

This confirms that the feasibility of vNOTES is limited in case of severe pelvic adhesions, or in those cases in which entrance to the Douglas pouch is not possible, such as in the presence of rectovaginal endometriosis and scars (8). Our experience highlights how a careful selection of patients is critical when using vNOTES. A bimanual rectovaginal examination before surgery should evaluate the anterior and posterior cul-de-sac for any nodularity, mass lesion, or scar tissue, which are contraindications for vNOTES. Also, a detailed history should be included, asking about endometriosis, pelvic inflammatory disease, and past pelvic surgery possible adhesions.

In our experience, the overall conversion rate to laparoscopy was 4.3% and it results in line with the conversion rate to laparotomy describe in the literature for TLH (Table 2) (19). No laparotomic conversion was needed in our experience.

Some concerns have been raised about the risk of post-operative wound infection when using a non-sterile entry (8, 16). Similar to Baekelandt et al. (14), Su et al. (16), and Wang et al. (13), in our case series, we did not observe any vaginal cuff or pelvic infection, even using three different antibiotic prophylaxis schemes. Lee et al. (15) described an overall incidence of pelvic infection of 3.1%; all the cases happened within the first 20 cases and the authors hypnotize that they may be due to unskilled technique of colpotomy and incomplete hemostasis. A subsequent review suggested that the vNOTES approach may reduce surgical wound infections compared to other laparoscopic techniques (12).

Moreover, avoiding abdominal port insertion may reduce post-operative pain, and other insertion site complications such as post-operative hernia, hematoma, subcutaneous emphysema, and vascular injuries. All these advantages should contribute to reducing the post-operative hospital stay (7). Between the studies that we evaluated for comparison, only Baekelandt et al. (14), provided a post-operative pain valuation. In our experience, the lack of abdominal wall incision had reduced pain and improved early mobility post-operatively. For our patients undergoing vNOTES, the mean post-operative VAS score at 24 h was 3.6. In the study by Baekelandt et al. (14), the mean post-operative VAS at 24 h was even significantly lower (mean 1.7). Other trials confirmed that vNOTES significantly reduce post-operative pain and may allow the treatment of a large number of patients in a day-care setting (7, 9). We observed that a significant number of patients were fit to go home on the same day (26.1%); however, due to our limited experience with this kind of surgery, we decide to favor discharge only from day 1. However, later in our experience, five patients (10.9%) went home on the same day, due to their optimal clinical conditions and the proximity of their residence to the hospital.

The potential impairment of sexual life due to the adoption of the transvaginal route had raised major concerns both in patients and surgeons. In a previous study on the female perception of transvaginal cholecystectomy, 68% and 43% of patients had worries regarding dyspareunia and decreased sensibility during intercourse, respectively (20). Notably, in a subsequent study on 220 women who underwent transvaginal cholecystectomy, 93% of the patients did not report any change in sexual function quality (21).

In vNOTES hysterectomy, the vaginal cuff is incised and closed as in vaginal hysterectomy and TLH, and we did not expect any difference in the incidence of dyspareunia. Anyway, our patients did not report chronic pelvic pain, dyspareunia, or impaired sexual satisfaction at 6-months follow-up. Our data are in line with the one provided by Lee et al. (15) and Baekelandt et al. (7) that reported no dyspareunia or impairment in sexual function at 3- and 6-months follow-up.

## Conclusion

In conclusion, vNOTES has proven to be a feasible and safe approach, and we believe that it could help to overcome some limitations of both conventional laparoscopic and vaginal surgery in selected women. We acknowledge that our study has some limitations as the population number and a non-comparative design. Moreover, we must acknowledge that all our centers are referral centers for gynecological surgery, and all the procedures were performed by experienced surgeons, proficient in both vaginal and laparoscopic surgeries. In future studies, we think it would be interesting to assess vNOTES learning curves for surgeons with different levels of experience.

However, we think that our initial experience could help clarify some issues with the adoption of vNOTES for the first time.

## Data availability statement

The raw data supporting the conclusions of this article will be made available by the authors, without undue reservation.

## Ethics statement

This study was carried out in agreement with the recommendations of the Good Clinical Practice (ICH/GCP), Ministerial Decree of 1997. All subjects gave written informed consent in accordance with the Declaration of Helsinki (clinical trial number 14354). The studies involving human participants were reviewed and approved by the Università degli Studi di Enna “Kore”. The patients/participants provided their written informed consent to participate in this study.

## Author contributions

TS, MLI, PS, and MM conceived and coordinated the project. TS, PS, MLI, MM, PM, BP, TB, and MC performed the surgical procedures. MLI, FM, MF, and GD collected the data and responsible for the data curation. FM and MLI prepared the first draft of the manuscript and responsible for the analysis and visualization of results. MLI, FM, TS, PS, and MM were involved in the interpretation of the data. TS revised it critically. All authors have given their approval for this version to be published.

## References

[1] Schlaerth AC Abu-Rustum NR. Role of minimally invasive surgery in gynecologic cancers. *Oncologist.* (2006) 11:895–901. 10.1634/theoncologist.11-8-895 16951393

[2] Aarts JW Nieboer TE Johnson N Tavender E Garry R Mol BWJ Surgical approach to hysterectomy for benign gynaecological disease. *Cochrane Datab Syst Rev.* (2015) 2015:CD003677. 10.1002/14651858.CD003677.pub5 26264829 PMC6984437

[3] Medeiros LR Stein AT Fachel J Garry R Furness S. Laparoscopy versus laparotomy for benign ovarian tumor: a systematic review and meta-analysis. *Int J Gynecol Cancer.* (2008) 18:387–99. 10.1111/j.1525-1438.2007.01045.x 17692084

[4] Kalloo AN Singh VK Jagannath SB Niiyama H Hill SL Vaughn CA Flexible transgastric peritoneoscopy: a novel approach to diagnostic and therapeutic interventions in the peritoneal cavity. *Gastrointest Endoscopy.* (2004) 60:114–7. 10.1016/S0016-5107(04)01309-4 15229442

[5] Reddy N Rao P. Per oral transgastric endoscopic appendectomy in human. In: *Proceedings of the 45th Annual Conference of the Society of Gastrointestinal Endoscopy of India.* Jaipur (2004).

[6] Autorino R Yakoubi R White WM Gettman M De Sio M Quattrone C Natural orifice transluminal endoscopic surgery (NOTES): where are we going? A bibliometric assessment: bibliometric assessment of notes. *BJU Int.* (2013) 111:11–6. 10.1111/j.1464-410X.2012.11494.x 23323699

[7] Baekelandt J De Mulder P Le Roy I Mathieu C Laenen A Enzlin P Hysterectomy by transvaginal natural orifice transluminal endoscopic surgery versus laparoscopy as a day-care procedure: a randomised controlled trial. *BJOG: Int J Obstet Gy.* (2019) 126:105–13. 10.1111/1471-0528.15504 30325565

[8] Li CB Hua KQ. Transvaginal natural orifice transluminal endoscopic surgery (vNOTES) in gynecologic surgeries: A systematic review. *Asian J Surg.* (2020) 43:44–51. 10.1016/j.asjsur.2019.07.014 31444108

[9] Yang YS Kim SY Hur MH Oh KY. Natural orifice transluminal endoscopic surgery-assisted versus single-port laparoscopic-assisted vaginal hysterectomy: a case-matched study. *J Minimally Invasive Gynecol.* (2014) 21:624–31. 10.1016/j.jmig.2014.01.005 24462594

[10] Wang X Li J Hua K Chen Y. Transvaginal natural orifice transluminal endoscopic surgery (vNOTES) hysterectomy for uterus weighing =1 kg. *BMC Surg.* (2020) 20:234. 10.1186/s12893-020-00897-3 33046022 PMC7552525

[11] Lauterbach R Matanes E Amit A Wiener Z Lowenstein L. Transvaginal natural orifice transluminal endoscopic (vNOTES) hysterectomy learning curve: feasibility in the hands of skilled gynecologists. *Isr Med Assoc J.* (2020) 22:13–6. 31927799

[12] Housmans S Noori N Kapurubandara S Bosteels JJA Cattani L Alkatout I Systematic review and meta-analysis on hysterectomy by vaginal natural orifice transluminal endoscopic surgery (vNOTES) compared to laparoscopic hysterectomy for benign indications. *JCM.* (2020) 9:3959. 10.3390/jcm9123959 33297354 PMC7762322

[13] Wang CJ Go J Huang HY Wu KY Huang YT Liu YC Learning curve analysis of transvaginal natural orifice transluminal endoscopic hysterectomy. *BMC Surg.* (2019) 19:88. 10.1186/s12893-019-0554-0 31291917 PMC6621966

[14] Baekelandt J. Total vaginal NOTES hysterectomy: a new approach to hysterectomy. *J Minimally Invasive Gynecol.* (2015) 22:1088–94. 10.1016/j.jmig.2015.05.015 26009278

[15] Lee CL Wu KY Su H Wu PJ Han CM Yen CF. Hysterectomy by transvaginal natural orifice transluminal endoscopic surgery (NOTES): a series of 137 patients. *J Minimally Invasive Gynecol.* (2014) 21:818–24. 10.1016/j.jmig.2014.03.011 24681063

[16] Su H Yen CF Wu KY Han CM Lee CL. Hysterectomy via transvaginal natural orifice transluminal endoscopic surgery (NOTES): Feasibility of an innovative approach. *Taiwanese J Obstetr Gynecol.* (2012) 51:217–21. 10.1016/j.tjog.2012.04.009 22795097

[17] Lee CL Wu KY Su H Wu PJ Han CM Wang CJ Natural orifice transluminal endoscopic surgery in gynecology. *Gynecol Minimally Invasive Ther.* (2012) 1:23–6. 10.1016/j.gmit.2012.08.007

[18] Alkatout I. Complications of laparoscopy in connection with entry techniques. *J Gynecol Surg.* (2017) 33:81–91. 10.1089/gyn.2016.0111 28663686 PMC5477171

[19] Twijnstra ARH Blikkendaal MD van Zwet EW Jansen FW. Clinical relevance of conversion rate and its evaluation in laparoscopic hysterectomy. *J Minimally Invasive Gynecol.* (2013) 20:64–72. 10.1016/j.jmig.2012.09.006 23312244

[20] Bucher P Ostermann S Pugin F Morel P. Female population perception of conventional laparoscopy, transumbilical LESS, and transvaginal NOTES for cholecystectomy. *Surg Endosc.* (2011) 25:2308–15. 10.1007/s00464-010-1554-4 21301884

[21] Mofid H Emmermann A Alm M von Waldenfels HA Felixmüller C Zornig C. Is the transvaginal route appropriate for intra-abdominal NOTES procedures? Experience and follow-up of 222 cases. *Surg Endosc.* (2013) 27:2807–12. 10.1007/s00464-013-2812-z 23392983

